# Logical gates in actin monomer

**DOI:** 10.1038/s41598-017-11333-7

**Published:** 2017-09-18

**Authors:** Andrew Adamatzky

**Affiliations:** University of the West of England, Bristol, BS16 1QY United Kingdom

## Abstract

We evaluate information processing capacity of a single actin molecule by calculating distributions of logical gates implemented by the molecule via propagating patterns of excitation. We represent a filamentous actin molecule as an excitable automaton network (F-actin automaton). where every atom updates its state depending on states of atoms its connected to with chemical bonds (hard neighbours) and atoms being in physical proximity to the atom (soft neighbours). A resting atom excites if a sum of its excited hard neighbours and a weighted sum of its soft neighbours belong to some specified interval. We demonstrate that F-actin automata implement OR, AND, XOR and AND-NOT gates via interacting patterns of excitation. Gate AND is the most common gate and gate XOR is the rarest. Using the architectures of gates discovered we implement one bit half-adder and controlled-not circuits in the F-actin automata. Speed and space values of the F-actin molecular computers are discussed.

## Introduction

Actin is a protein presented in all eukaryotic cells in forms of globular actin (G-actin) and filamentous actin (F-actin)^1–3^. G-actin, polymerises in double helix of filamentous actin, during polymerisation G-actin units slightly change their shapes and thus become F-actin units^4^. The actin networks play a key role in information processing^5–8^ in living cells. In ref.^9^ we proposed a model of actin polymer filaments as two chains of one-dimensional binary-state semi-totalistic automaton arrays and uncovered rules supporting traveling localisations, discrete analogs of ionic waves proposed in actin filaments^10^. We speculated that a computation in actin filaments could be implemented with travelling patterns of excitation. We implemented computing schemes in several families of actin filament models, from quantum automata to lattice with Morse potential^11–15^. The models proposed previously dealt with a coarse-grain computation on actin filaments, chains of F-actin units, where each monomer stored a single bit. A density of computing elements in actin-based computers could be substantially increased if we implement several logical gates in a single F-actin monomer. Thus, to evaluate information processing capacity of an actin molecule we decided to calculate distributions of logical gates implemented by the molecule via propagating patterns of excitation. The approach was successfully tested on automaton model of verotoxin protein^16^ and scoping experiments have been conducted on actin molecular^17^.

The paper is structured as follows. In Sect. 2 we design an actin molecule model in automaton network where nodes takes excited, refractory and resting states and update their states depending on states of their neighbours. The logical gates, Sect. 4, are discovered by selecting a pair of inputs node at random, exciting them with all possible combinations of binary inputs, recording states of all other nodes in the molecular automaton and selecting combinations of input and output bodes that implement desired logical functions. The gates are cascaded in a one-bit half-adder, Sect. 5, and controlled-not gate, Sect. 6. In Sect. 7 we estimate densities of logical gates in actin and speed of computation.

## Methods

**Figure 1:**
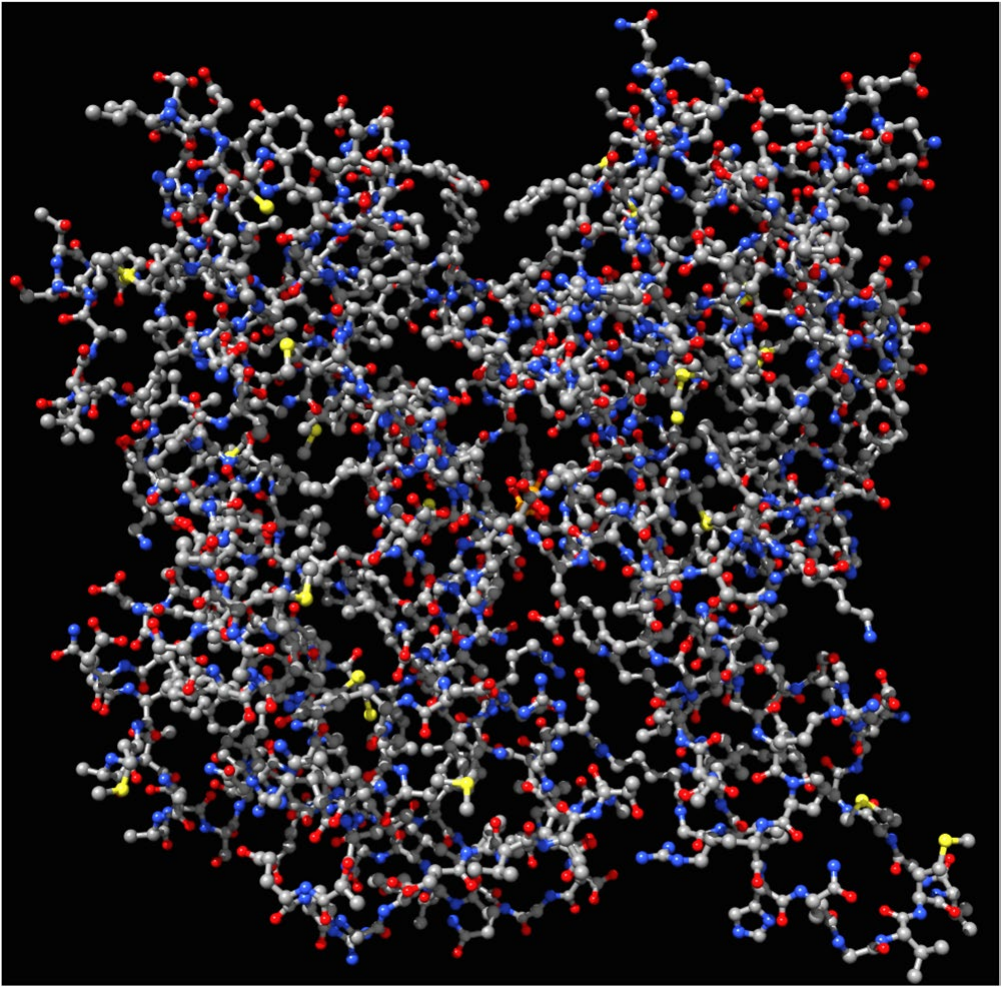
F-actin molecule, CPK colouring, the strucutre of the molecule is determined in ref.^4^.

We use a structure of F-actin molecule produced using X-ray fibre diffraction intensities obtained from well oriented sols of rabbit skeletal muscles^4^. The structure was calculated with resolution 3.3 Å in radial direction and 5.6 Å along the axis, see illustration of the molecular structure in Fig. 1. The molecular structure is converted to a non-directed graph ${\mathscr{A}}$ embedded in Euclidean space: where every node represents an atom and an edge corresponds to a bond between the atoms. F-actin is represented by a graph ${\mathscr{A}}=\langle {\bf{V}},{\bf{E}},{\bf{C}}\rangle$, where **V** is a set of nodes, **E** is a set of edges, **C** is a set of Euclidean coordinates of nodes form **V**.

The graph ${\mathscr{A}}$ has 2961 nodes, 3025 edges. Minimum degree is 1, maximum is 4, average is 2.044 (with standard deviation 0.8) and median degree 2. There are 883 nodes with degree 1, 1009 nodes with degree 2, 1066 nodes with degree 3 and two nodes with degree 4. The graph ${\mathscr{A}}$ has a diameter (longest shortest path) 1130 nodes, and a mean distance (mean shortest path between any two nodes) 376, and a median distance 338.

Let *u*(*s*) be nodes of ${\mathscr{A}}$ that connected with an edge with a node *s*, they correspond to atoms connected with a bond to atom *s*. We call them hard-neighbours because their neighbourhood is determined by chemical structure of F-actin. Actin molecule is folded in 3D. Let *δ* be an average distance between two hard-neighbours, for F-actin *δ* = 1.43 units. Let *w*(*s*) be nodes of ${\mathscr{A}}$ that are at distance not exceed *ρ*, in Euclidean space, from node *s*. We call them soft neighbours because their neighbourhood is determined by 3D structure of F-actin. Thus each node *s* has two neighbourhoods:

1. hard neighbourhood *u*(*s*) = {*p* ∈ **V** : (*sp*) ∈ **E**} (actin automata with hard neighbourhood were firstly proposed by us in ref.^17^), and
2. soft neighbourhood $w(s)=\{p\in {\bf{V}}\,:\,p\notin u(s)\,{\rm{and}}\,d({c}_{s},\,{c}_{p})\le \rho \}$, where *d*(*c*_*s*_, *c*_*p*_) is a distance between nodes *s* and *p* in 3D Euclidean space and *c*_*s*_, *c*_*p*_ ∈ **C**.

**Figure 2:**
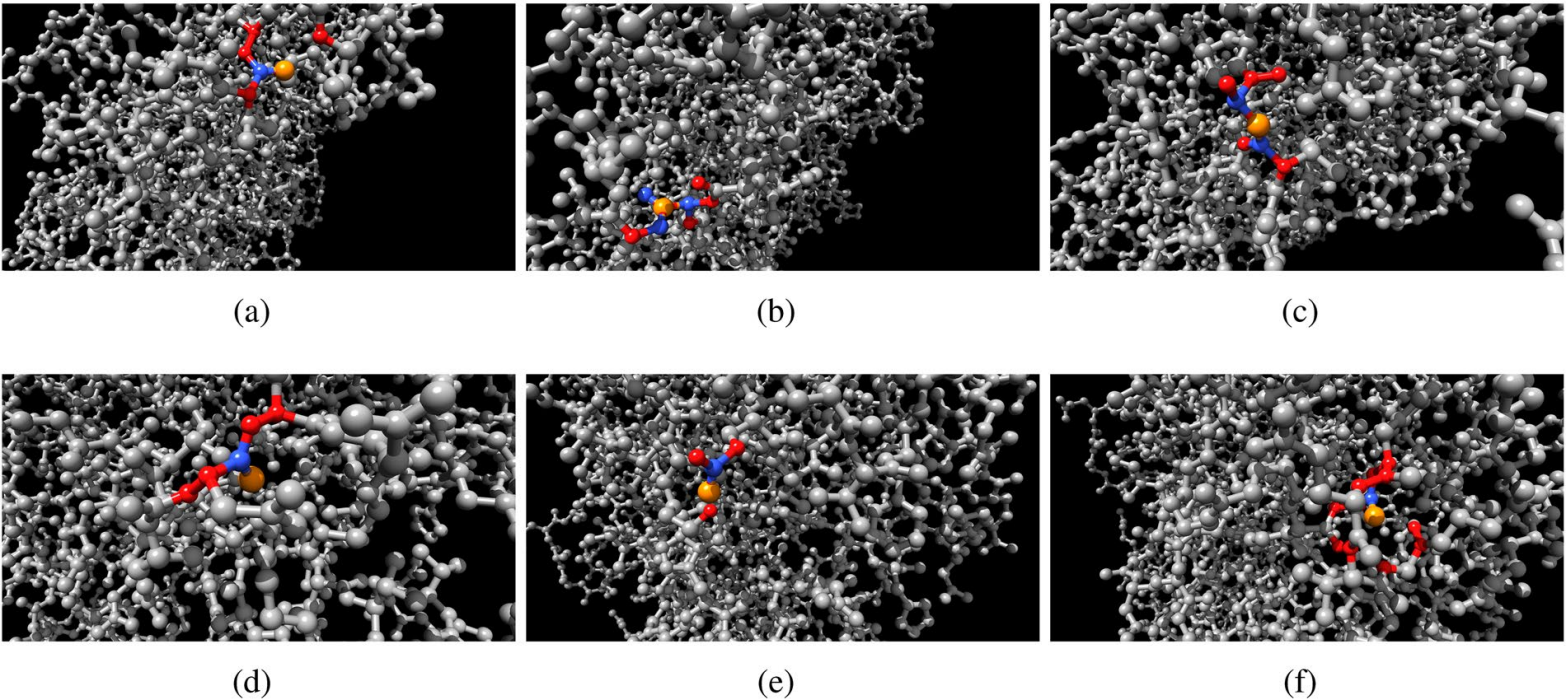
Examples of neighbourhoods, central node is orange, hard neighbours *u*(*s*) are red, soft neighbours, for *ρ* = 3, are blue *w*(*s*).

**Figure 3:**
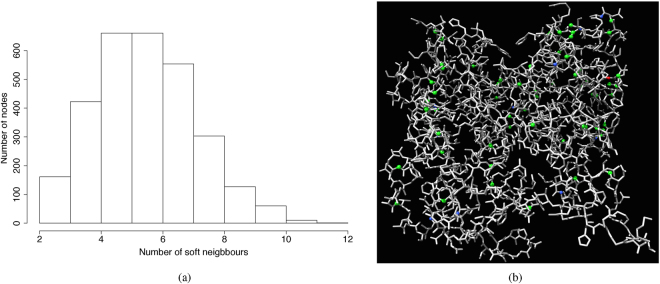
Soft neighbourhood. (**a**) Distribution of soft neighbourhood sizes for *ρ* = 3. (**b**) Nodes with maximum number of soft neighbours are coloured: red (12 neighbours), blue (10 neigbours), green (9 neighbours).

**Figure 4:**
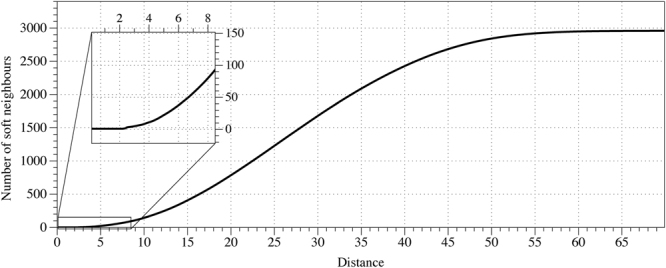
Number of soft neighbours of a node versus distance *ρ*, averaged over all nodes.

Distribution of hard neighbourhood sizes corresponds to distribution of degrees of the nodes. Distribution of soft neighbourhood sizes is determined by distance *ρ* (Fig. 4). We have chosen *ρ* = 3 so size of *w*(*s*) does not substantially exceeds size of *u*(*s*) (Fig. 3a). Examples of neighbourhoods are shown in Fig. 2, a soft neighbour of a node *s* is not necessarily a hard neighbour of the *s*’s neighbour. Distribution of nodes with highest numbers of soft neighbours is shown in (Fig. 3b).

Each node *s* of ${\mathscr{A}}$ takes three states: resting ($\circ$), excited ($\star$) and refractory ($\bullet$). A resting node *s* ^*t*^ = $\circ$ excites depending on a number ${\sigma }_{s}^{t}$ of its excited neighbours in neighbourhoods *u*(*s*) and *w*(*s*): ${\sigma }_{s}^{t}={\sum }_{p\in u(s)}\,\{{p}^{t}=\star \}$ $\mu \,{\sum }_{p\in w(s)}\,\{{p}^{t}=\star \}\,$ $+\,\mu \,{\sum }_{p\in w(s)}\,\{{p}^{t}=\star \}$. We consider values of *μ* = 0.2, 0.4, 0.8 and $\mu =\frac{\delta }{d({c}_{s},\,{c}_{p})}$. An excited node takes refractory state. A refractory node takes resting state. We consider two types of excitation. In threshold excitation a resting node *s* excites if a ${\sigma }_{s}^{t}$ exceeds threshold *θ*:

1 $${s}^{t+1}=\{\begin{array}{cc}\star , & {\rm{i}}{\rm{f}}\,\,{\sigma }_{s}^{t} > \theta \\ \bullet , & {\rm{i}}{\rm{f}}\,\,{s}^{t}=\star \\ \circ , & {\rm{o}}{\rm{t}}{\rm{h}}{\rm{e}}{\rm{r}}{\rm{w}}{\rm{i}}{\rm{s}}{\rm{e}}\end{array}$$

In interval excitation a resting node *s* excites if ${\sigma }_{s}^{t}$ belongs to a discrete interval Θ = [*θ*_1_, *θ*_2_], i.e. a set of discrete numbers from *θ*_1_ to *θ*_2_:

2 $${s}^{t+1}=\{\begin{array}{cc}\star , & {\rm{i}}{\rm{f}}\,\,{\sigma }_{s}^{t}\in {\rm{\Theta }}\\ \bullet , & {\rm{i}}{\rm{f}}\,\,{s}^{t}=\star \\ \circ , & {\rm{o}}{\rm{t}}{\rm{h}}{\rm{e}}{\rm{r}}{\rm{w}}{\rm{i}}{\rm{s}}{\rm{e}}\end{array}$$

We search for the Boolean logical gates by selecting a pair of nodes from **V**, exciting these nodes with combinations of inputs 01, 10, 11 (where ‘0’ is a logical False and ‘1’ is logical True) and checking states of all other nodes.

Let two nodes *i* and *j* be selected as inputs and one node *p* as an output. Boolean logical variables are *x* and *y* (inputs) and *z* (output). Initially automaton ${\mathscr{A}}$ is in its resting state. Logical values of inputs are converted to initial states of input nodes as follows. If *x* = 1 then ${s}_{i}^{0}=\star$, if *y* = 1 then ${s}_{j}^{0}=\star$.

We allow the automaton to evolve till a limit cycle or a globally resting state are reached. During the automaton’s evolution we monitor state of the output node *p*. If at some time step *t* we have ${s}_{p}^{t}=\star$ we assign *z* = 1. For every rule of excitation, and exact parameters, we performed random trials, by selecting 5 · 10^4^ pairs of nodes at random, exciting them with strings $(\circ \star )$, $(\star \circ )$, $(\star \star )$, and recording outputs at all other nodes of ${\mathscr{A}}$.

For modelling and analyses we used Processing, R, and iGraph. Patterns of excitation dynamics are visualised in Chimera.

## Dynamics of Excitation

**Figure 5:**
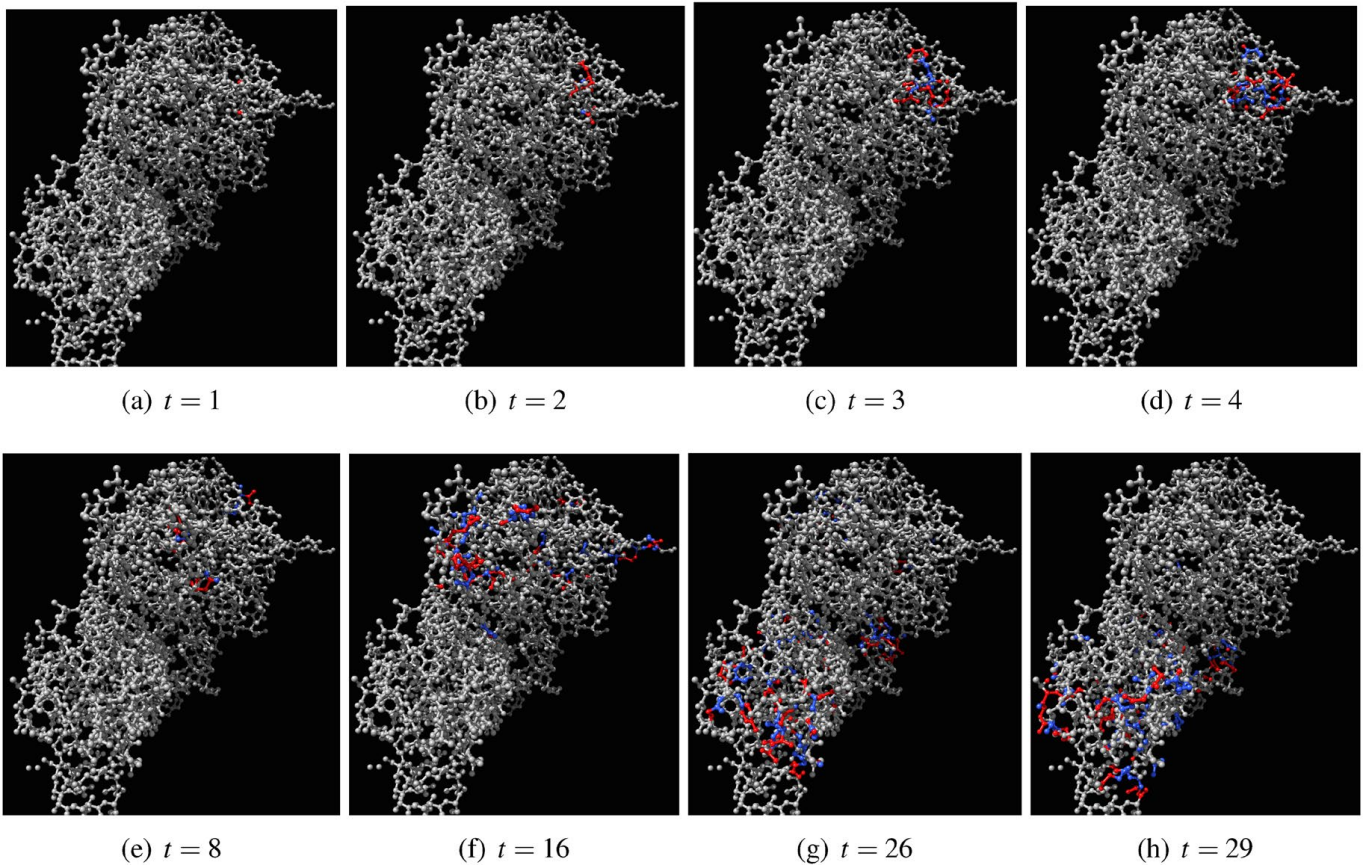
Evolution of automaton ${\mathscr{A}}$ governed by Eq. 1, *θ* = 0, *μ* = 0.2. Two nodes of the resting automaton are excited initially, *t* = 1. Excited nodes are red, refractory are blue, resting are grey.

**Figure 6:**
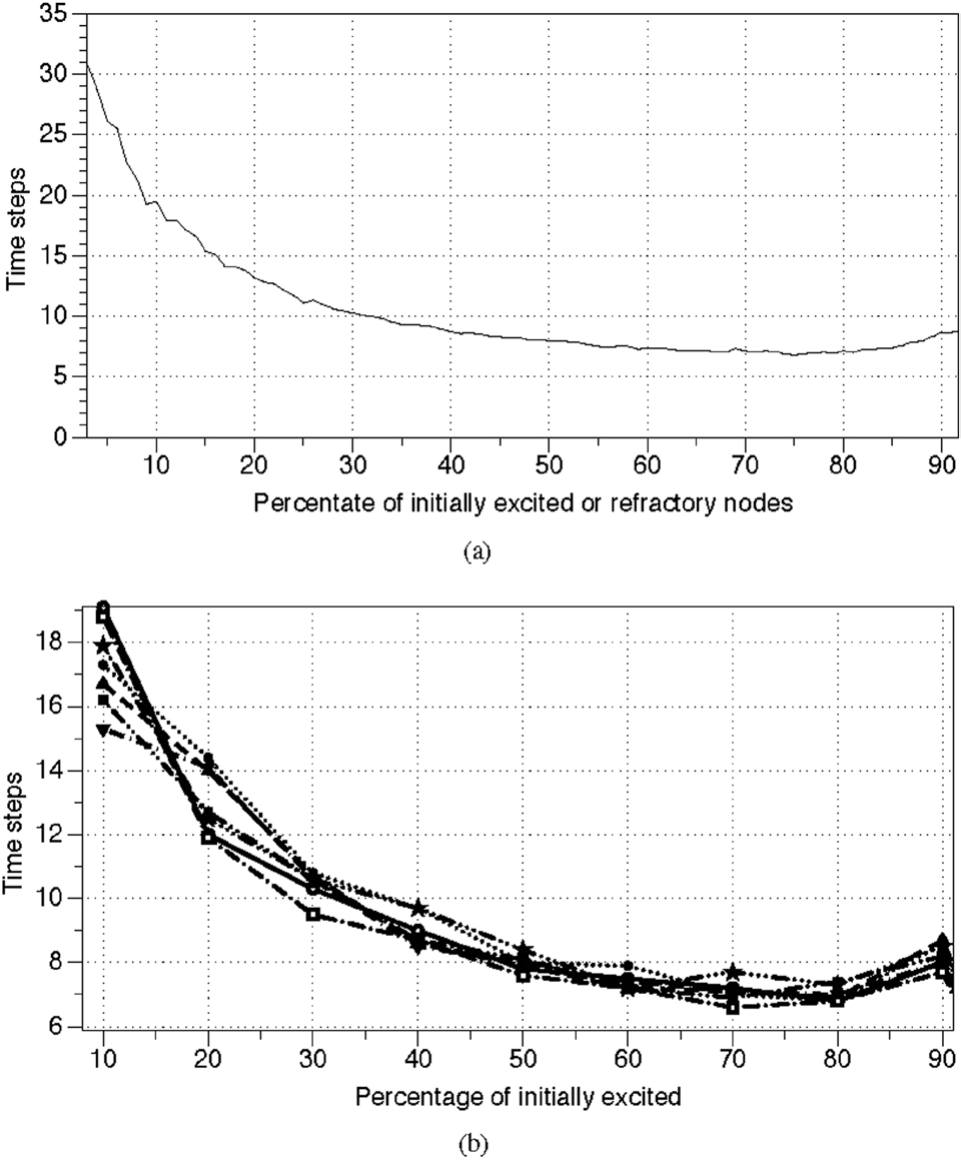
Transient periods. (**a**) Average transient period of ${\mathscr{A}}$ governed by Eg. 1, *θ* = 0, *μ* = 0.2, depending on a number of initially excited or refractory nodes. Percentage of initially excited nodes ranges from 1 to 100, with increment 1. For each percentage we conducted 100 trials. (**b**) Average transient periods of ${\mathscr{A}}$ governed by Eg. 1 calculated in ten trials for several values *μ*, depending on a number of initially excited or refractory nodes. The values *μ* are as follows *μ* = 0.001, circle; *μ* = 0.05, solid disc; *μ* = 0.1, triangle up; *μ* = 0.2, triangle down; *μ* = 0.3, empty square, *μ* = 1, solid square, *μ* = 5, empty star.

We studied excitation dynamics of actin automata with only hard neighbourhoods, see ref.^17^, thus here we discuss dynamics of automata with hard and soft neighbourhoods. When a small number of nodes of otherwise resting automaton ${\mathscr{A}}$ governed by Eq. 1, *θ* = 0, *μ* = 0.2, is excited, the wave of excitation propagates in the automaton similarly to classical excitation waves in a non-homogeneous medium. Excitation travels omnidirectional, albeit with distortions determined by geometry of the F-actin molecule (Fig. 5). Eventually wave-fronts of the excitation wave collide with each other and annihilate in the result of the collision. The automaton returns to its resting state. A number of excited nodes necessary to kick start the excitation increases with increase of *θ*. Typically, when *θ* exceeds 1 no random pattern of excitation leads to a sustainable propagation excitation waves. If ${\mathscr{A}}$, governed by Eq. 1, is initially perturbed by assigning some pool of nodes either excited or refractory states the automaton evolves to short limit cycles, typically with period of three time steps. Length of transient period versus number of initially stimulated nodes is shown in Fig. 6a, it decreases with increasing size of an initially perturbed domains. The dependence is observed for various values of *μ* > 0 (Fig. 6b).

**Figure 7:**
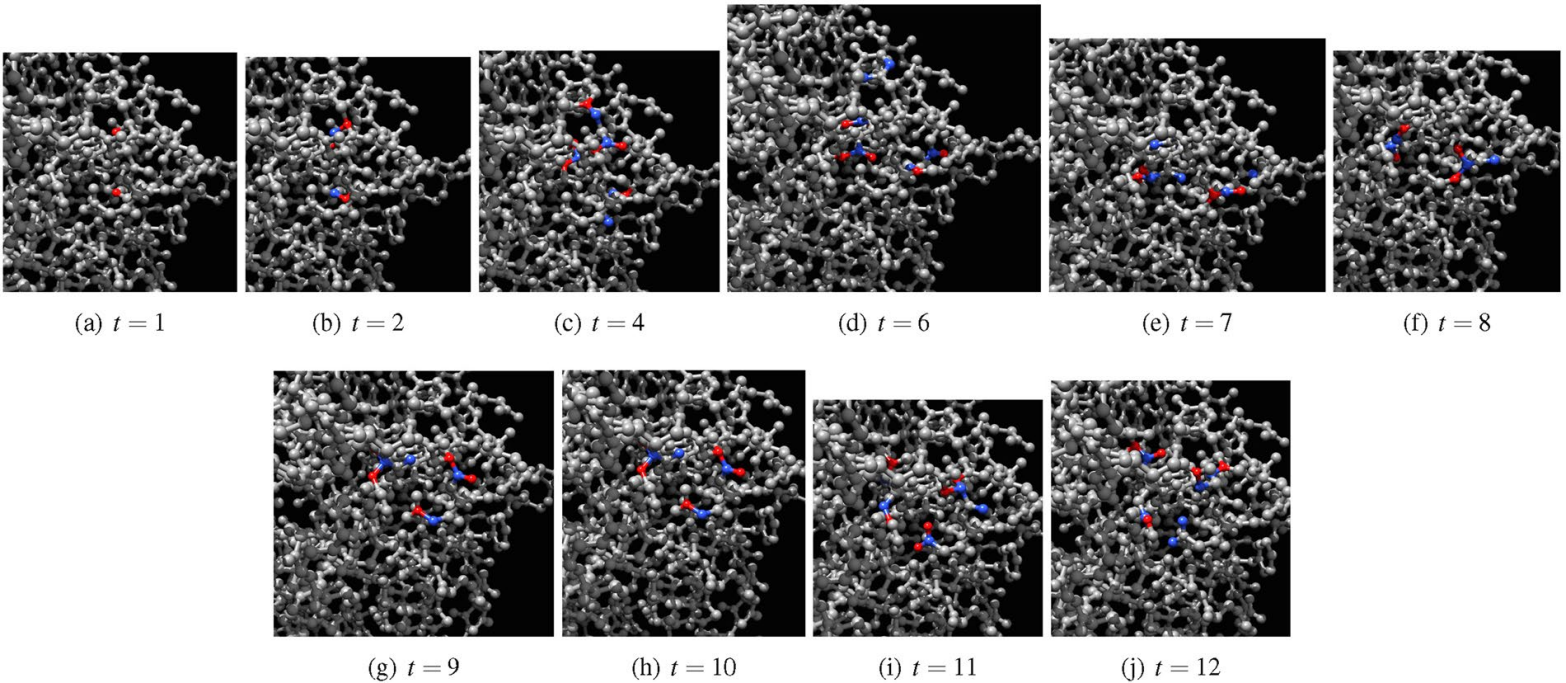
Evolution of automaton ${\mathscr{A}}$ governed by Eq. 2, Θ = $[1]$, *μ* = 0.2. Two node of the resting automaton are excited initially, *t* = 1. Excited nodes are red, refractory are blue, resting are grey.

**Figure 8:**
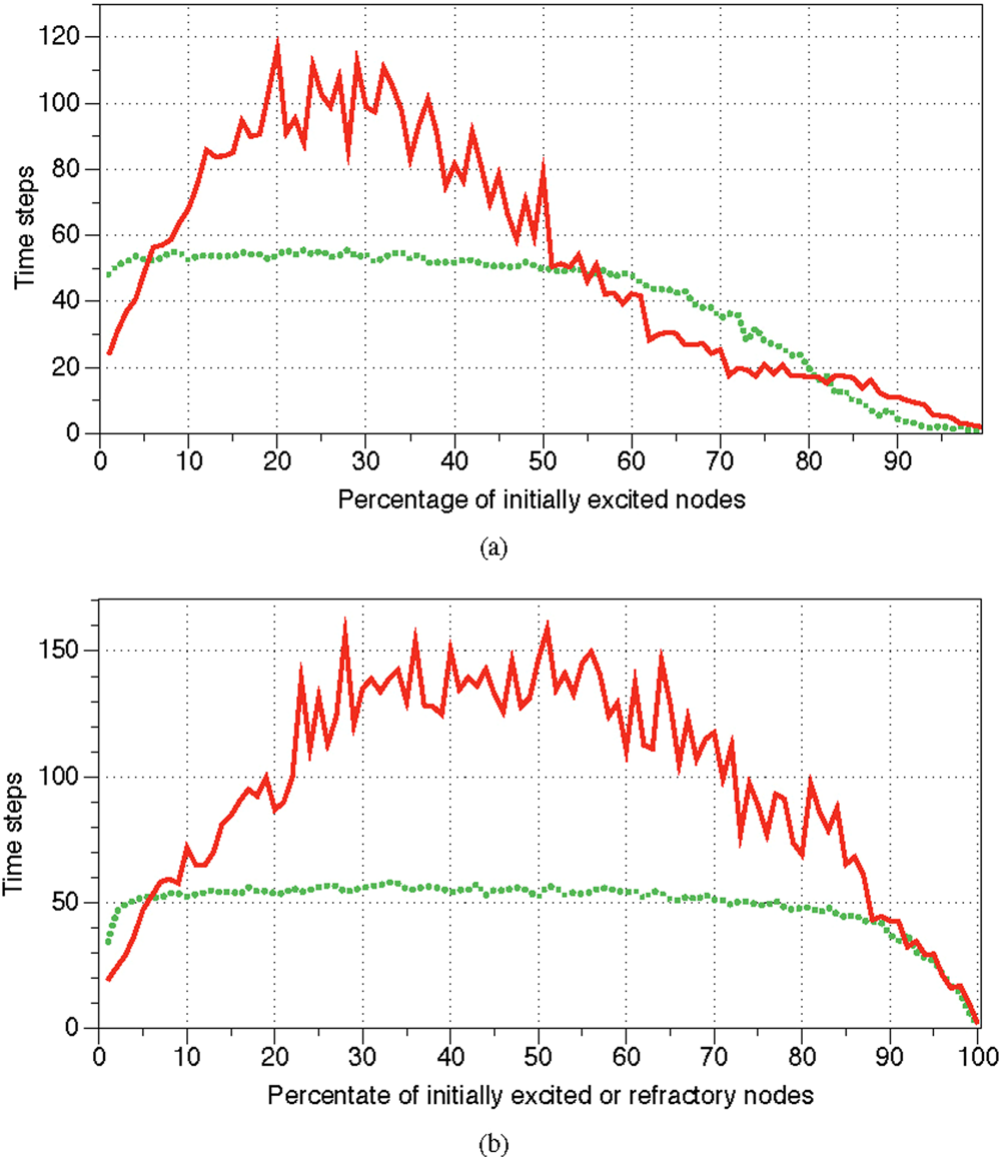
Lengths of transient periods (solid red) and of limit cycles (dotted green) of automaton ${\mathscr{A}}$, governed by Eq. 2, Θ = $[1]$, *μ* = 0.2, dependent on a number of initially stimulated nodes. (**a**) A percentage of nodes is initially excited. (**b**) A percentage of nodes is initially excited or made refractory. Percentage of stimulated nodes ranges from 1 to 95, with increment 1. For each percentage we conducted 100 trials.

A stimulated resting automaton ${\mathscr{A}}$ governed by Eq. 2, Θ = $[1]$, *μ* = 0.2 always ends its evolution in one of limit cycles, where excitation travels along some finite cyclic paths (Fig. 7). A length of an average limit cycle of ${\mathscr{A}}$ governed by Eg. 2 does not depend on a number of initially stimulated (made of excited or refractory) nodes. The length is around 50 time steps when a percentage of initially perturbed nodes is between 10% and 90% (Fig. 8). Transient period *p*, expressed in time steps, shows quadratic dependence on a percentage of initially stimulated nodes *ρ*: *p* = 23.24 + 4.9185*ρ* + (−0.051891)*ρ* ^2^ (Fig. 8).

## Boolean Gates

Typically, gates discovered in ${\mathscr{A}}$ have two inputs nodes and several nodes which represent the same output.

Automaton ${\mathscr{A}}$ with threshold excitation rule Eq. (1), *θ* = 0, implements AND and AND-NOT gates, and also identity gates where output represents a value of one of the inputs(Table 1). Almost every pair of nodes selected has some output nodes which represent these gates (Fig. 1). No XOR gate was found in ${\mathscr{A}}$ governed by rule (1) with or without soft neighbours, therefore we will not study this particular automaton further.

Automata governed by interval excitation rule Eq. (2) realise input selector gates (*z* = *x* and *z* = *y*), OR, AN, XOR and AND-NOT ($z=\overline{x}y$ and $z=x\overline{y}$) gates (Table 2). Frequencies of gates discovered are characterised in Table 2. Frequencies of gates are similar for automata where a sum of excited soft neighbours are weighted by distance between a node and its soft neighbour $\mu =\frac{\delta }{d({c}_{s},{c}_{p})}$ (Table 2a) and automata where weights of the soft neighbours are fixed to *μ* = 0.2 (Fig. 2b) or *μ* = 0.4 (Table 2c). The frequencies of gates are also robust with respect to a size of excitation interval: compare Θ = $[1]$ (Table 2b,c) and Θ = $[1,\,2]$ (Table 2d). Majority of gates discovered are input selector gates, they can be found in a half of trials. Second most common gate is and. Gates AND-NOT and XOR are much less frequent that other gates, with XOR gate being the rarest one, typically found just in one of thousand trials. Thus a hierarchy of frequencies of gates is AND > OR > AND-NOT > XOR. We can see a role of soft neighbours in formation of and gates by comparing (Table 2e) with (Table 2a–d). In the automaton without soft neighbours, *μ* = 0, input selector gates are rare, XOR gates are discovered in half of the trials and OR and AND-NOT gates are discovered in almost all trials.

**Table 1:** Statistics of two-inputs one-output gates discovered in threshold excitation ${\mathscr{A}}$ without soft neighbours, rule Eq. (1), *μ* = 0.2, *θ* = 0.

	*x* + *y*	${\boldsymbol{x}}\overline{{\boldsymbol{y}}}$	$\overline{{\boldsymbol{x}}}{\boldsymbol{y}}$
*m*	0.9997	0.9965	0.9936
*n*	2370	278	277
st. dev. *n*	152	116	118

The table shows average ratio *m* of input pairs of nodes, i.e. a number of nodes detected in all samples divided by a number of samples, and average number *n* of output nodes per pair of input nodes, with its standard deviation. The gates are discovered in 50 K trials. Identity gates are not shown.

**Table 2:** Statistics of two-input one-output gates discovered in interval excitation ${\mathscr{A}}$, rule Eq. (2).

	*x*	*y*	*x* + *y*	*xy*	*x* ⊕ *y*	$x\overline{y}$	$\overline{x}y$
(a)							
*m*	0.41	0.40	0.006	0.025	0.001	0.003	0.003
*n*	34	33	27	6	5	10	6
st. dev. *n*	30	33	20	3	3	8	2
(b)							
*m*	0.41	0.42	0.007	0.022	0.001	0.004	0.004
*n*	34	34	27	6	25	14	17
st. dev *n*	30	30	25	7	28	18	20
(c)							
*m*	0.42	0.42	0.006	0.022	0.001	0.003	0.004
*n*	34	34	26	6	28	15	15
st. dev *n*	30	30	24	7	30	19	17
(d)							
*m*	0.42	0.42	0.007	0.02	0.001	0.004	0.004
*n*	34	33	24	6	17	14	16
st. dev *n*	30	30	21	5	23	17	20
(e)							
*m*	0.06	0.05	0.9	0	0.5	0.9	0.9
*n*	2141	2193	2803	0	2	40	39
st. dev *n*	1260	1236	12	0	2	9	9

The table shows average ratio *m* of input pairs of nodes, i.e. a number of nodes detected in all samples divided by a number of samples, and average number *n* of output nodes per pair of input nodes, with its standard deviation. The gates are discovered in 50 K trials. Identity gates are not shown. (a) $\mu =\frac{\delta }{d({c}_{s},{c}_{p})}$, Θ = $[1]$. (b) *μ* = 0.2, Θ = $[1]$. (c) *μ* = 0.4, Θ = $[1]$. (d) *μ* = 0.2, Θ = $[1,\,2]$. (e) *μ* = 0, Θ = $[1]$.

**Figure 9:**
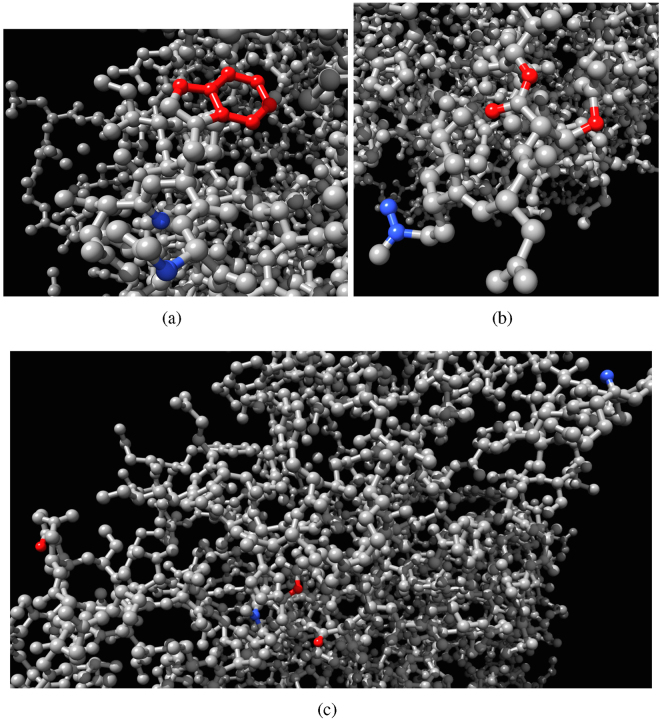
Architectures of XOR (**a**,**b**) gates, two examples, and AND (**c**) gate realised in ${\mathscr{A}}$ rule Eq. (2), *μ* = 0.2. Input nodes are blue, output nodes are red.

Exemplar architectures of XOR and AND gates are shown in Fig. 9. In XOR gates input nodes are located in close proximity of each other (Fig. 9a) or even hard neighbours (Fig. 9b). The output nodes of XOR gate exhibit a similar degree of geographic proximity, e.g. output nodes in Fig. 9a are atoms of the same aromatic ring. Configuration of gate and presents a different picture: distance between input and outputs nodes are large, sometimes half of the graph diameter (Fig. 9c).

## One-bit half adder

A one-bit half-adder is a gate with two inputs *x* and *y* and two outputs *xy* (sum) and *x* ⊕ *y* (carry). To evaluate if ${\mathscr{A}}$ implements a half-adder we must search for two inputs nodes for which there are at lest two output nodes: one node outputs AND and another node outputs XOR. In 5 ⋅ 10^3^ samples of automaton ${\mathscr{A}}$ governed by rule Eq. (2), *μ* = 0.2, Θ = $[1]$, we found just a single pair of nodes producing AND and XOR. In the same sampling size of automaton governed by rule Eq. (2), *μ* = 0.2, Θ = $[1,\,2]$, we found two pairs of input nodes which has output nodes producing XOR and AND.

**Figure 10:**
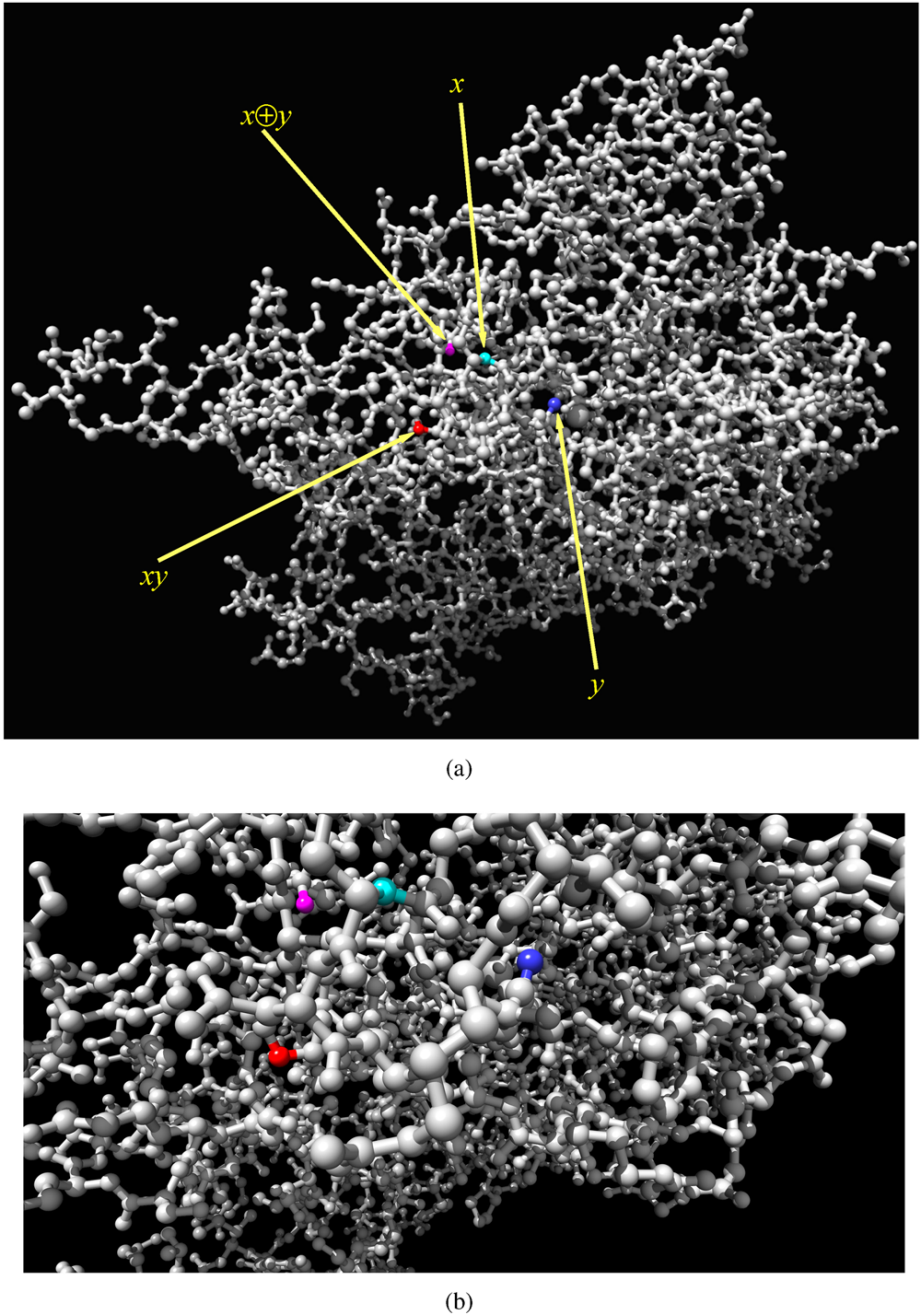
Architecture of a one-bit half-adder implemented in ${\mathscr{A}}$, governed by rule Eq. (2), *μ* = 0.2. Input nodes *x* and *y* are coloured cyan and blue. Output node *xy* is red and output node *x* ⊕ *y* is magenta. (**a**) Positions of input and output nodes with respect to the whole molecule. (**b**) Zoomed configuration.

**Figure 11:**
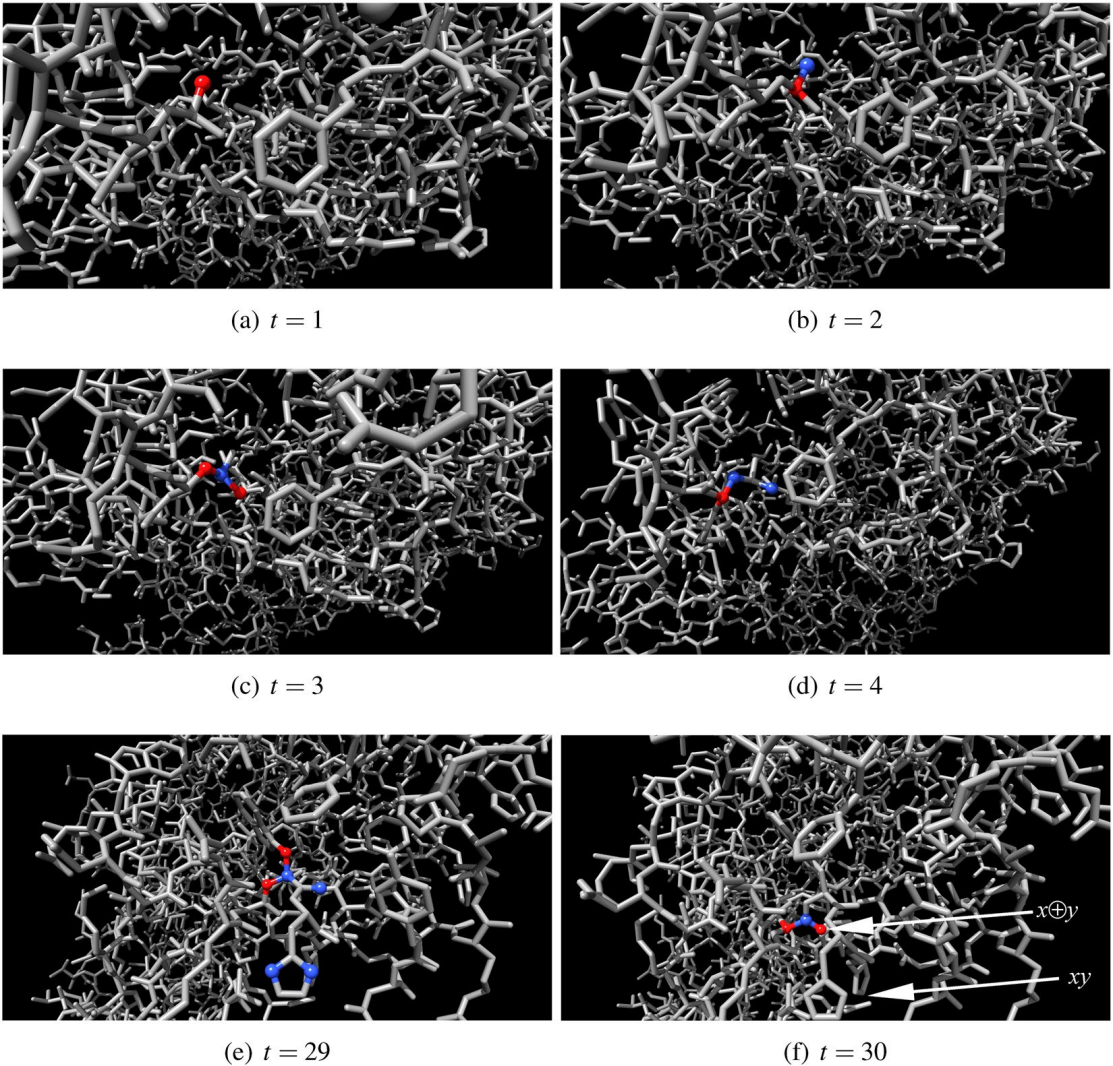
One-bit half-adder in action. Inputs are *x* = 0 and *y* = 1. Output nodes *x* ⊕ *y* and *xy* are indicated by arrows in (**f**). Output node *xy* is in resting state, output node *x* ⊕ *y* is excited. Excited nodes are red, refractory are blue.

**Figure 12:**
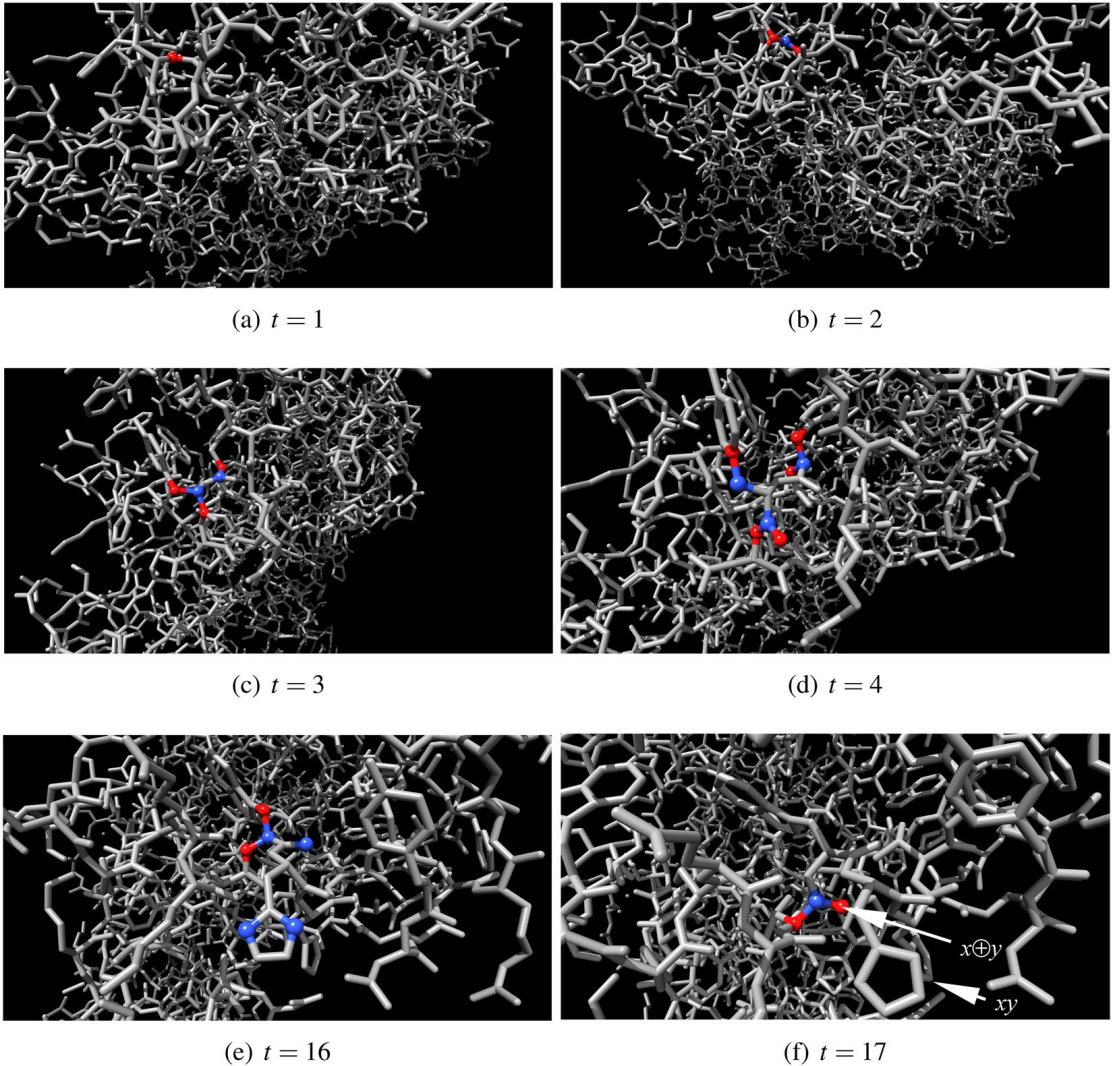
One-bit half-adder in action. Inputs are *x* = 1 and *y* = 0. Output nodes *x* ⊕ *y* and *xy* are indicated by arrows in (**f**). Output node *xy* is in resting state, output node *x* ⊕ *y* is excited. Excited nodes are red, refractory are blue.

**Figure 13:**
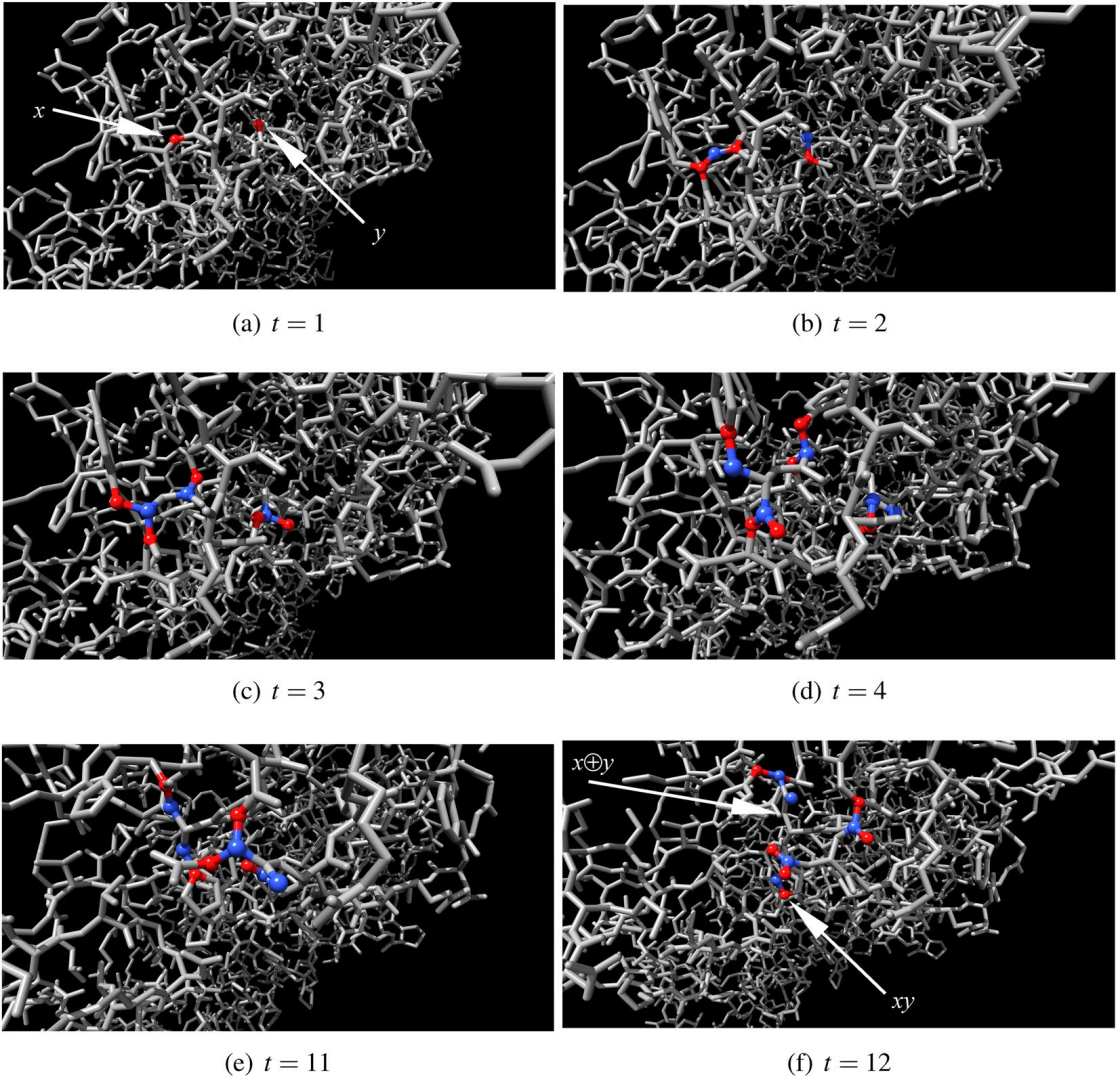
One-bit half-adder in action. Inputs are *x* = 1 and *y* = 1. Input nodes are indicated by arrows in (**a**), they both are excited at *t* = 1. Output nodes *x* ⊕ *y* and *xy* are indicated by arrows in (**f**). Output node *xy* is excited, output node *x* ⊕ *y* is in resting state. Excited nodes are red, refractory are blue.

**Figure 14:**
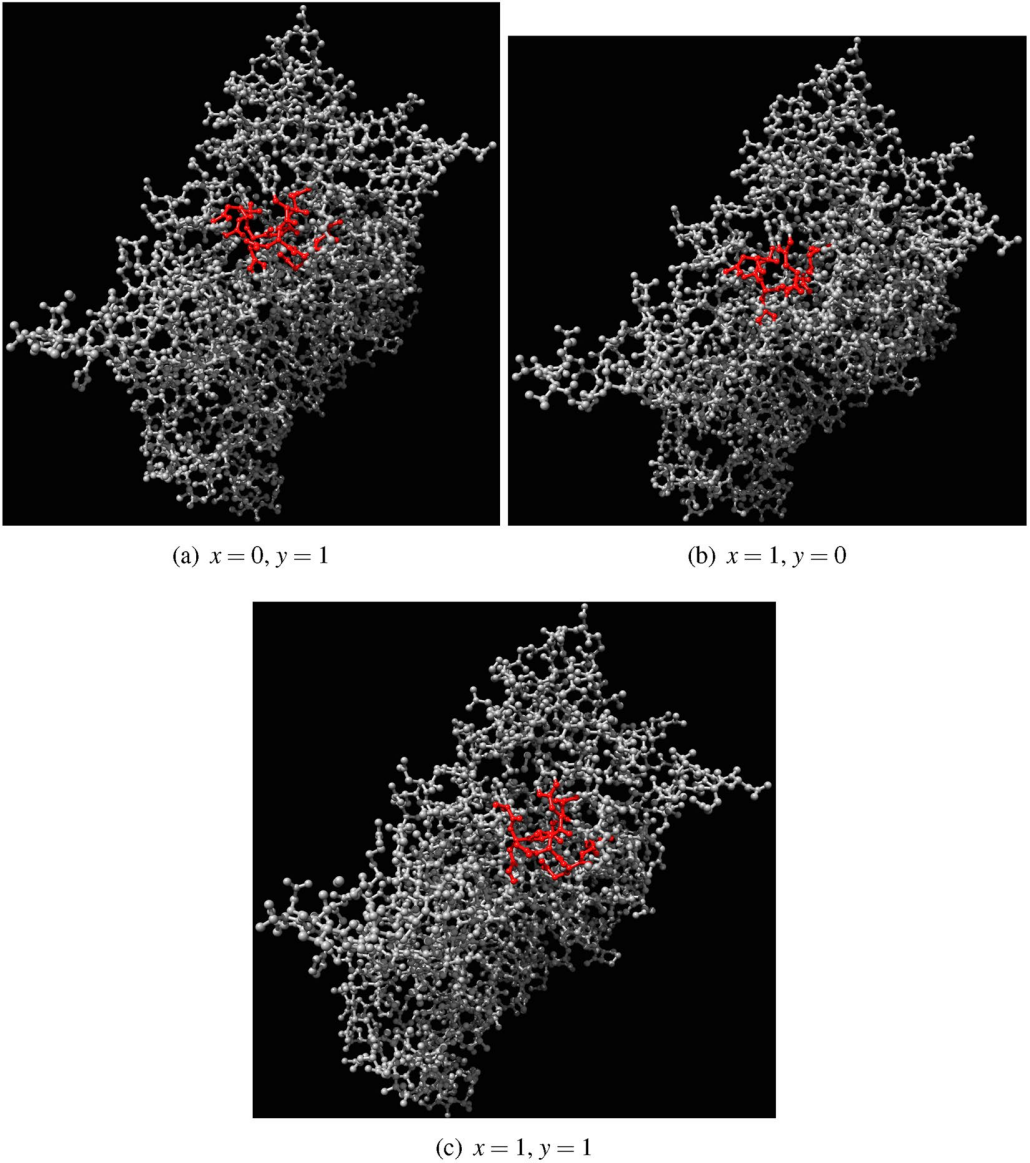
Excitation paths in half-adder for inputs (**a**) *x* = 0, *y* = 1, (**b**) *x* = 1, *y* = 0, (**c**) *x* = 1, *y* = 1. Nodes which have been excited at least once during the automaton evolution, for given states of input nodes, are coloured red.

Architecture of an exemplar half-adder is shown in Fig. 10. Atoms IDs and names are as follows: *x* (ID = 696, N atom in tyrosine), *y* (ID = 731, CD1 atom in leucine), *xy* (ID = 677, NE2 atom in histamine), *x* ⊕ *y* (ID = 639, O atom in isoleucine). Dynamics of excitation in the half-adder is shown for inputs *x* = 0 and *y* = 1 (Fig. 11), *x* = 1 and *y* = 0 (Fig. 12) and *x* = 1 and *y* = 1 (Fig. 13). Traces of excitation are shown in Fig. 14.

## Controlled-not gate

**Figure 15:**
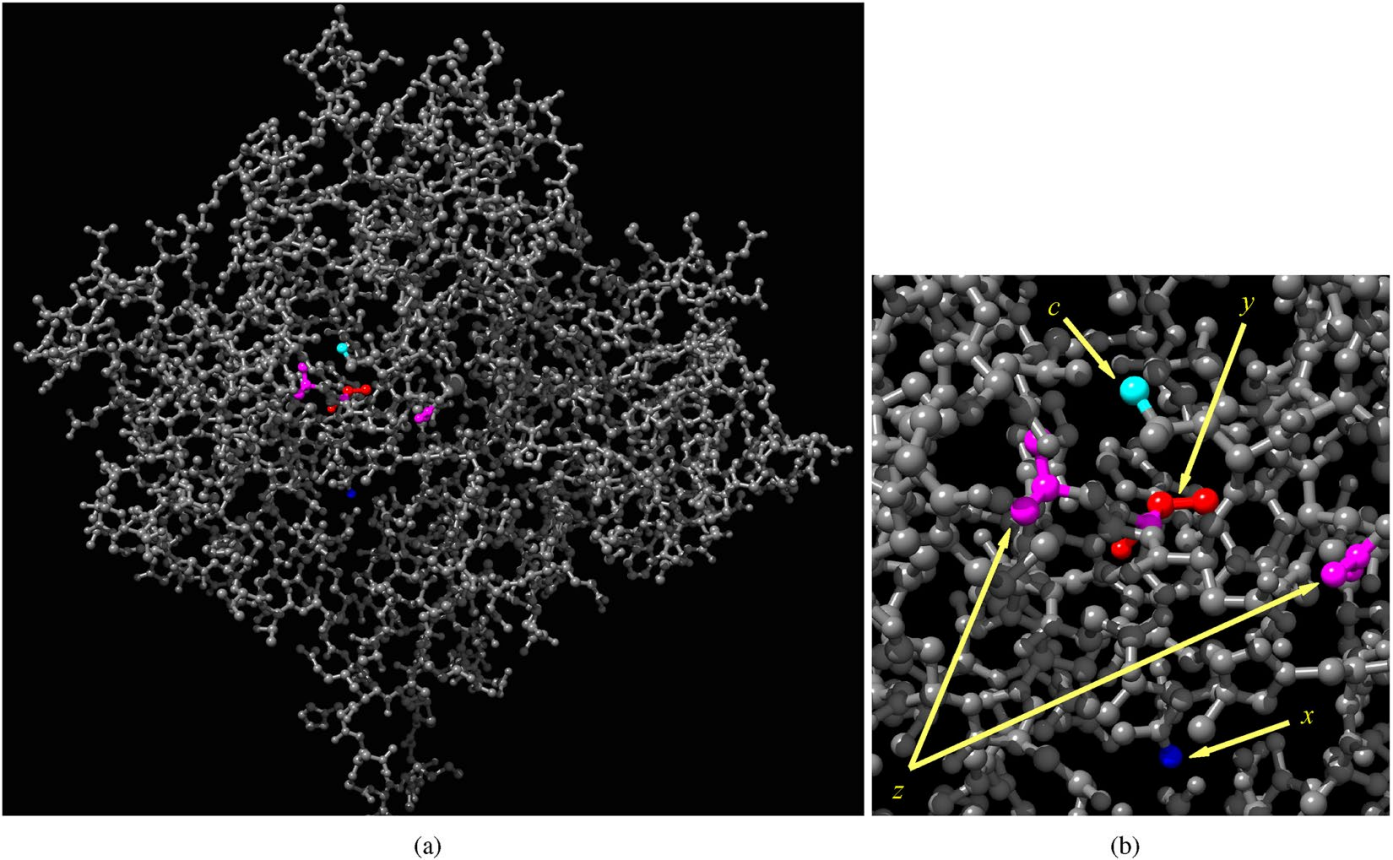
An example of cnot gate. (**a**) Location of the gate in the molecule. (**b**) Zoomed architecture. Cyan node is a control input *c*, blue node is a target input *x*, each of pink nodes represents the same output *z*, and any of red nodes is an output *y*.

**Figure 16:**
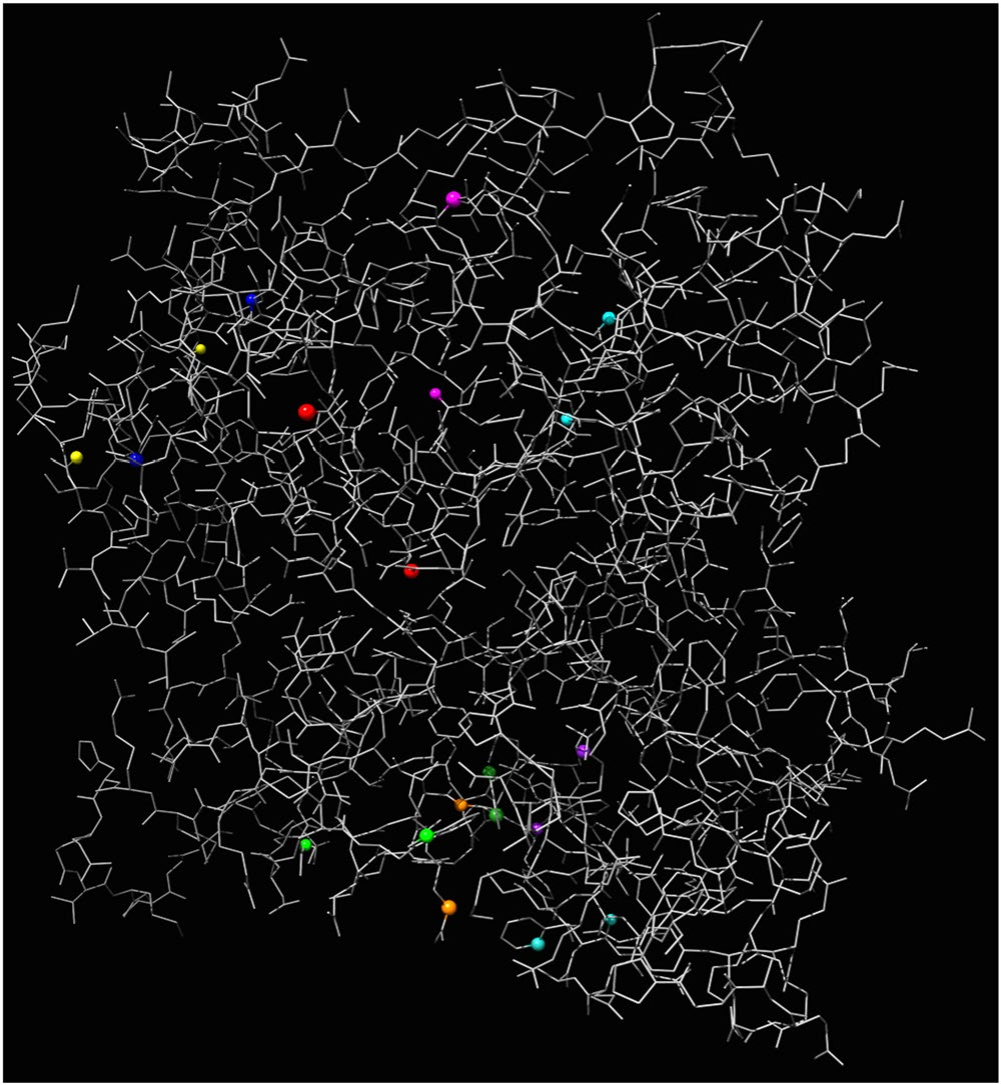
Eleven pairs of input nodes of CNOT gates. Each pair of nodes has its unique colour.

Controlled not gate firstly appeared in Toffoli’s paper on reversible computing^18^, the gate was coined ‘controlled not’ gate by Feynman^19^. Let us call the gate cnot. The cnot gate has two inputs *x* and *c* and two outputs *y* and *z*: *z* = *c*, and *y* = *x* if *c* = 0 and $y=\overline{x}$ if *c* = 1; alternative, *y* = *x* ⊕ *c*. By sampling 5 · 10^4^ pairs of nodes in ${\mathscr{A}}$ governed by rule Eq. (2), *μ* = 0.2, Θ = $[1]$, we found ten pairs of nodes (*x*, *c*), which have outputs nodes acting as control *z* and target *y* (these pairs of input nodes are shown in Fig. 16). Configuration of one of the cnot gates is shown in Fig. 15. Output nodes lie between input nodes. Nodes outputting *z* = *c* are in two clusters, divided *y* nodes.

## Discussion

There are nearly 9 · 10^6^ pairs of inputs in an F-actin molecule. We sampled 5 · 10^4^ pairs of nodes and discovered that several species of logical gates can be realised by interacting patterns of excitation in F-actin. We considered two classes of excitation rules: threshold excitation and interval excitation. Threshold excitation automata realise only input selectors, OR and AND-NOT gates, thus posing a limited interest for future computing applications. In the rest of the paper we focused on interval excitation automata. Input selector gates, where output takes a value of one of the inputs, are most common. They found almost in half of the samples. The next most frequently found gate is AND. It is frequency is around 0.02. The rarest gate is XOR, frequency 0.001. Frequencies of AND-NOT gates are 0.003–0.004.

A range of excitation intervals Θ depends on a number of hard and soft neighbours. Number of hard neighbours is limited to 2–3 in average, number of soft neighbours depends on parameter *ρ* (Fig. 4). We studied automata with excitation intervals Θ = $[1]$ and Θ = $[1,\,2]$, other configurations of the excitation interval can be analysed in future, e.g. Θ = $[2,\,3]$ or Θ = $[1,\,3,\,4]$, etc., we do not expect thought that a distribution of logical gates discovered will be substantially affected by exact configuration of excitation interval. Soft neighbourhood of some nodes might be changed dynamically when conformation changes of the molecule are induced. Thus, in principle, we can tune configurations of logical circuits by controlling shape of the F-acting unit; this could be a topic of further studies.

How fast would be an F-actin computing unit if implemented in experimental laboratory conditions? An excitation in a molecule takes place when an electron in a ground state (analog of automaton resting state $\circ$) absorbs a photon and moves up to high yet unstable energy level (analog of automaton excited state $\star$). Later the electron returns to its ground state (transition period to the ground state is analogous to automaton refractory state $\bullet$). When returning to the ground state the electron releases photon which travels with speed 3 × 10^18^ Å per second. Assuming at each step of modelling excitation wave-front travels *ρ* = 3 Å, one step of automaton evolution equates to 10^−18^ sec, i.e. one attosecond, of real time. The half-adder produces results in at most 30 steps of automaton evolution (Fig. 11), i.e. in 30 attoseconds. That is upper boundary of operating frequency of actin-based computing devices will be 30 PetaHz. The outputs of the F-actin unit can be measured using controlled light waves and pulse trains^20–23^, or in case of using less exotic devices, train of 10^3^ impulses of the same data inputs can be sent to F-actin unit and output recorded via accumulating, e.g. capacitive, devices.

What if we were unable to excite a single node in F-actin unit? Then we could use larger probes. Will these affect frequency distribution of gates realisable in F-actin units? To find out we conducted 5 · 10^4^ trials, in each trial we selected a pair of nodes and, for inputs ‘1’, stimulated all nodes in a sphere radius 10 Å around the input node. Output nodes usually form clusters exceeding 10 Å, so we should care about probes only in inputs. Frequencies of logical gates found are shown in Table 3. By comparing Table 3 with frequencies of gates realisable during single node stimulation (Table 2d) we find that probe based inputting increases a number of gates discovered: AND-NOT gate by c. 18 times, OR gate by c. 7 times, XOR gate by c. 5 times.

**Table 3:** Frequencies of gates realisable in ${\mathscr{A}}$ governed by rule Eq. (2), *μ* = 0.2 and Θ = $[1,\,2]$.

	*x*	*y*	*x* + *y*	*xy*	*x* ⊕ *y*	${\boldsymbol{x}}\overline{{\boldsymbol{y}}}$	$\overline{{\boldsymbol{x}}}{\boldsymbol{y}}$
*m*	0.622	0.640	0.050	0.033	0.004	0.074	0.070
*n*	52	50	15	27	19	30	39
st. dev *n*	44	41	19	26	35	30	35

Inputs, for values ‘1’, are excited by probes of radius 10 Å. *m* is a ratio of input node pairs in the sample, i.e. a number of nodes detected in all samples divided by a number of samples, *n* is a number of output nodes for each pair of input nodes, and its standard deviation.

To evaluate density of gates we assume there are at most c. 8 · 10^6^ pairs of nodes in a F-actin molecule. Gate XOR has the lowest frequency 0.001 (Table 2d). Based on the frequency of XOR gate we assume there are at least 6 · 10^3^ XOR gates in a single F-actin molecule. Maximum diameter of an actin filament is 8 nm^24,25^. An actin filament is composed of overlapping units of F-actin. Thus, diameter of a single unit is c. 4 nm. There are estimated 6 K gates per 4 nm, or in terms of conventional circuits, when projected onto a 2 × 2 nm square, we have c. 16 · 16^18^ gates per sq inch.

Let us estimate temporal resources on the binary arithmetical computation realisable by F-actin molecular computer. In random samples of 5 · 10^4^ pairs of inputs nodes we found one one-bit half-adder. Taking lower boundary of pairs available as 8 · 10^6^, there are c. 160 one-bit half-adders in F-actin molecule, with possibility of constructing 80 one-bit full adders. Thus we can assume that at least 64-bit full adder can be constructed in a single F-actin unit. A 64-bit addition will take c. 1920 attoseconds, i.e. c. 2 femtoseconds.

Is a frequency distribution of logical gates a protein specific? Can such a distribution play a role of a fingerprint? Extensive analysis will be done in future. Right know we have data on just two protein molecules: actin (present paper) and verotoxin^16^. Let us do some comparisons. Actin automata governed by rule Eq. (1) with *θ* = 0 and *μ* = 0.2 implement 1.05 times more AND-NOT gates than verotoxin automata for the same rule and set of parameters. However, AND-NOT gates implemented in actin have 1.3 less average number of potential output nodes than that in verotoxin. When actin and verotoxin automata are governed by rule Eq. (2) with Θ = $[1]$ and *μ* = 0.2 actin implements three times more OR gates, the same number of and hates, five times more XOR gates and twice more AND-NOT gates than verotoxin does. With regards to average number of potential output nodes per input pair, actin has the same number for OR gate, c. 1.6 times less for XOR gate then verotoxin. Thus, actin molecule potentially implements more logical gates than verotoxin molecule however number of potential output nodes is less in actin.
